# Challenges Faced in Treating Maxillary Second Premolars With Three Canals: A Case Report

**DOI:** 10.7759/cureus.61391

**Published:** 2024-05-30

**Authors:** Khyati Manik, Anuja Ikhar, Aditya Patel, Manoj Chandak, Joyeeta Mahapatra, Jay Bhopatkar

**Affiliations:** 1 Department of Conservative Dentistry and Endodontics, Sharad Pawar Dental College and Hospital, Datta Meghe Institute of Higher Education and Research, Wardha, IND

**Keywords:** three canals, endodontic treatment, anatomic variation, maxillary premolar, endodontics

## Abstract

The maxillary second premolar has long been regarded as a tooth with a straight root canal anatomy, typically featuring a single root with two canals. However, emerging evidence suggests this tooth may harbor a more intricate internal morphology, challenging conventional perceptions, and treatment approaches. One such variation is three root canals within the upper second premolar, which has been gaining increasing attention in endodontic literature. Root canal treatment of maxillary second premolars with three canals demands meticulous debridement, shaping, and disinfection. A combination of magnification, illumination, and appropriate instrumentation aids in locating, negotiating, and cleaning the accessory canals effectively. Thorough cleaning of accessory canals can be achieved through effective irrigation solutions such as sodium hypochlorite, ethylenediaminetetraacetic (EDTA), and chlorhexidine, which help dissolve organic tissues and remove debris. Techniques like ultrasonic and sonic activation, as well as negative pressure irrigation systems, enhance the penetration and effectiveness of these irrigants. Additionally, the use of modern nickel-titanium rotary files, ultrasonic irrigation, and supplementary chelating agents enhances the management of complex canal configurations.

## Introduction

Root canal anatomy has long been a subject of fascination and complexity within dentistry. While much attention has been devoted to understanding the intricate canal configurations of molars, premolars have often been considered relatively straightforward. The comprehensive information about the morphology of the root and its canal, and the diagnosis and interpretation of the X-rays that are taken preoperatively, are prerequisites for root canal treatment. Many failures in the field of endodontics are caused by an inability to detect canals [1]. The doctor must be knowledgeable about all the variations in the root canals of every tooth.

The first upper premolar of the permanent dentition is characterized by two cone-shaped roots i.e. one palatal and one buccal root, where there can be a fusion of roots with a proper demarcation among them [2]. A 1% to 5% incidence rate of dividing the buccal root into two causes the tooth to have three canals in the two roots, of the upper first premolar [3]. Despite the challenges associated with the complex morphologies of the different canals of the root, the rotary nickel-titanium (Ni-Ti) endodontic file system enables the preparation and shaping of the root canal faster than traditional hand files [4]. 

In 0% to 6% of cases, upper first premolars with three roots are found, and typically, each root contains one canal. It's very uncommon for upper second premolars to have three roots and three canals [5]. A poorly documented case is reported [6-11], highlighting that an upper second premolar with three canals has an incidence of 1% according to Vertucci [12], and 0.3% according to Pecora et al. [13]. This report describes three canals in the two roots of the upper second premolar.

## Case presentation

**Figure 1:**
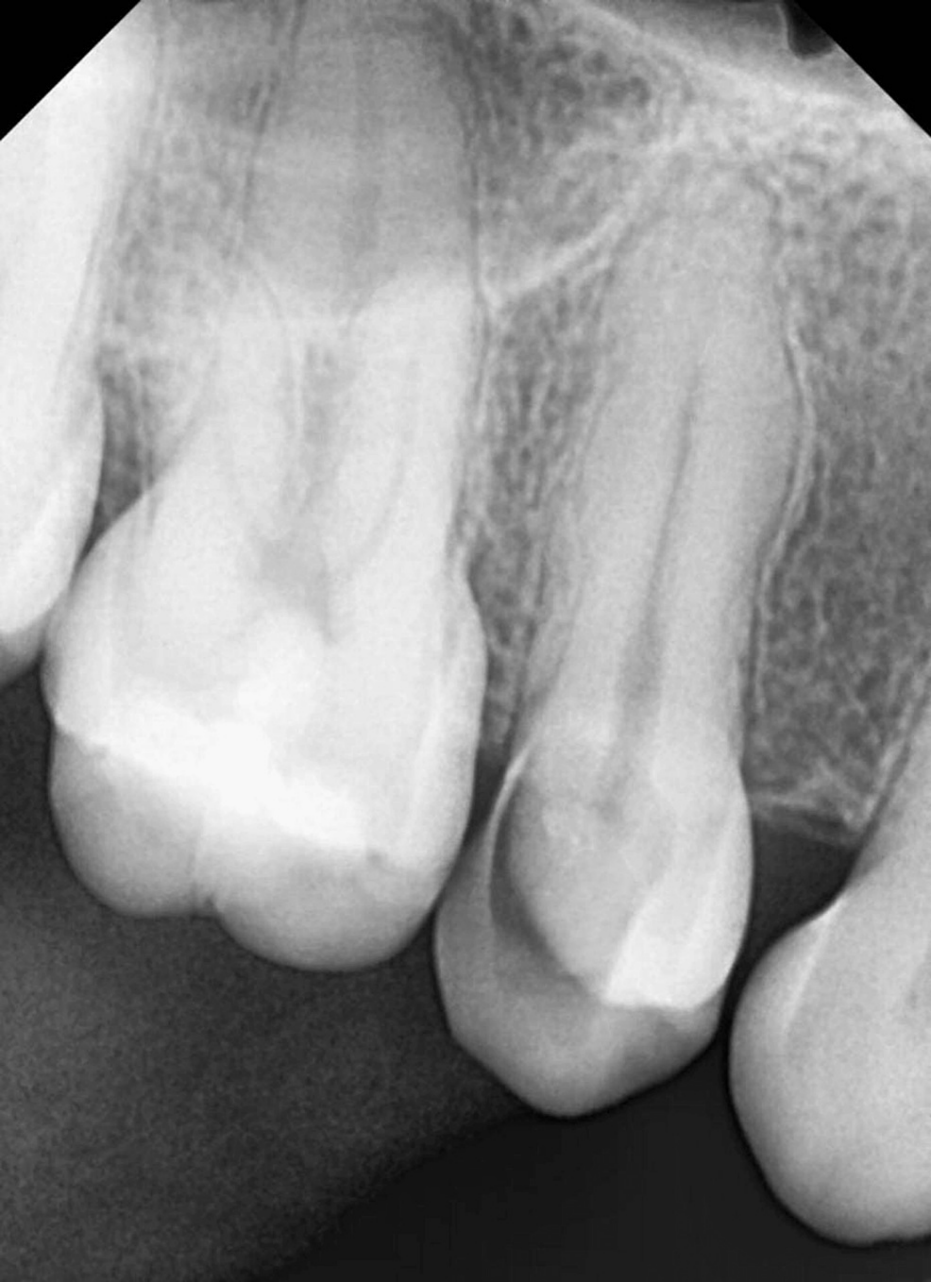
Preoperative radiograph

A 38-year-old male patient contacted the Endodontic Department of the Sharad Pawar Dental College and Hospital, Wardha, India, complaining of pain in the lower right jaw for the past month. When the chief complaint was specified, the pain was spontaneous and throbbing, persisted for minutes after removing the stimulus (usually heat) and was relieved by taking the medication (paracetamol 500 mg). Spontaneous episodes occurred throughout the night, disrupting sleep, and daily activities. The pain was sharp, severe, and difficult for the patient to localize to a specific tooth. The past medical history, as well as the past dental history, of the patient was non-significant. On clinical examination, distoproximal caries with the upper right second premolar was present, tenderness on vertical percussion was present, and no mobility was noted. There was no history of fever, swelling, or sinus associated with it. On radiographic examination, the radiolucency involving enamel, dentin, and pulp, as well as widening of the periodontal ligament space, was observed with the right upper second premolar tooth. Pulp neural sensibility tests, such as electric pulp test and thermal test, were performed. After performing an electric pulp test, a delayed response (Reading 38) was observed in the right upper second premolar compared to the contralateral left upper second premolar tooth (Reading 14). Following a hot gutta-percha test, where a 0.06 #15 gutta-percha (Dentsply Sirona, Charlotte, USA) was heated to 65.5°C and placed over the middle third of the buccal cusp for less than five seconds, the right upper second premolar responded with lingering pain upon removal of the stimulus. A diagnosis of “Symptomatic Irreversible Pulpitis with Apical Periodontitis” was made for the right upper second premolar (Figure 1).

**Figure 2:**
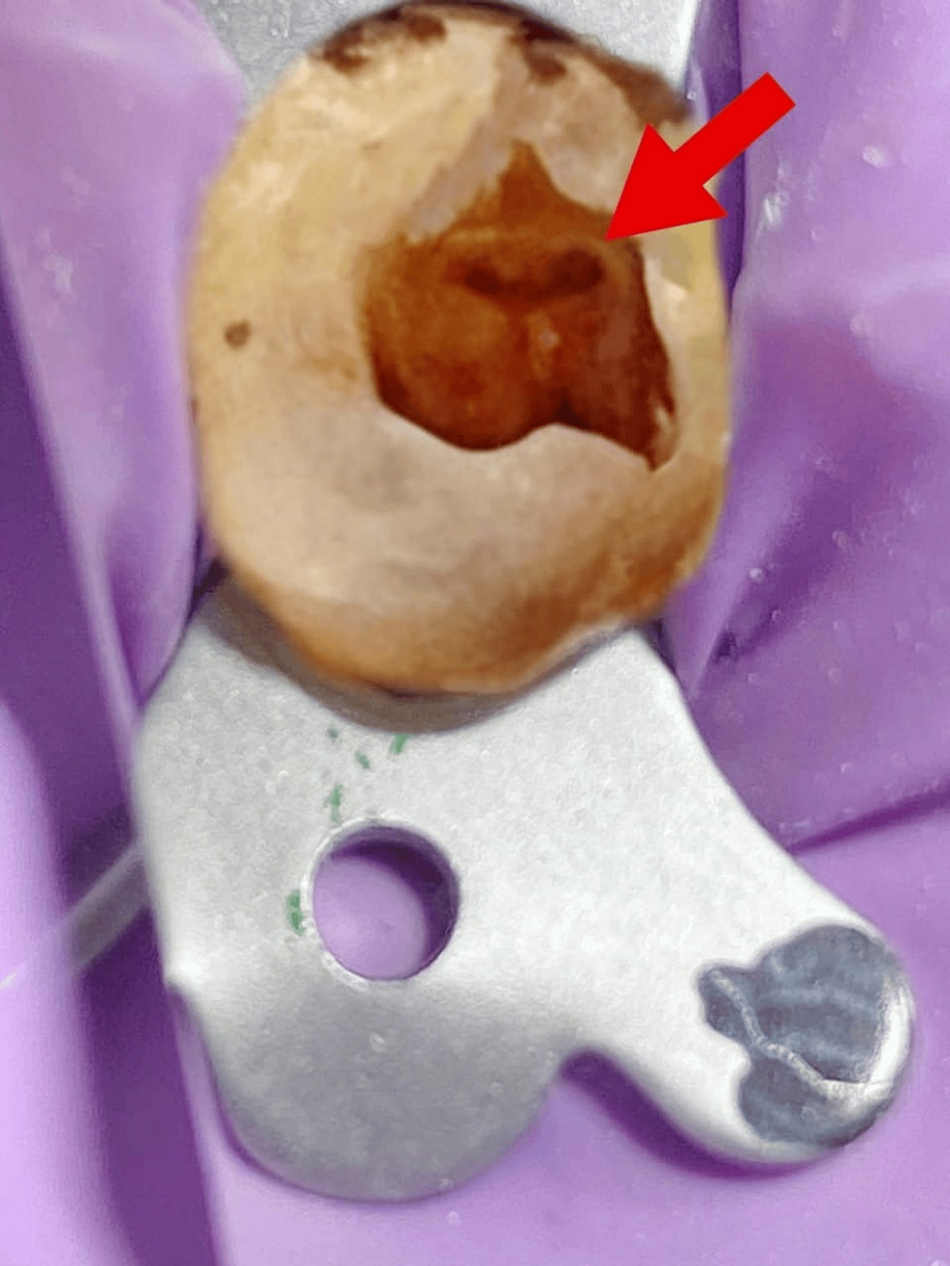
Access opening

The patient received 2% Xylocaine with 1:80,000 adrenaline (Xylocaine 2% Injection; Zydus Cadila, Ahmedabad, India). Rubber dam isolation was done. Round BR-45 (Mani, Inc., Utsunomiya, Japan) was used to prepare the access cavity, and safe end bur EX-24 (Mani, Inc., Utsunomiya, Japan) was used for the extension of the cavity in lateral motion. Once the pulp tissue was completely removed from the pulp chamber, two openings were seen i.e. mesiobuccal and palatal located below the buccal and palatal cusps, and a distinct opening was seen in the distal direction, which was found to be a distobuccal opening. The shape of the access cavity preparation was then modified to a 'T' shape to properly locate all three root canal openings of the upper second premolar (Figure 2).

**Figure 3:**
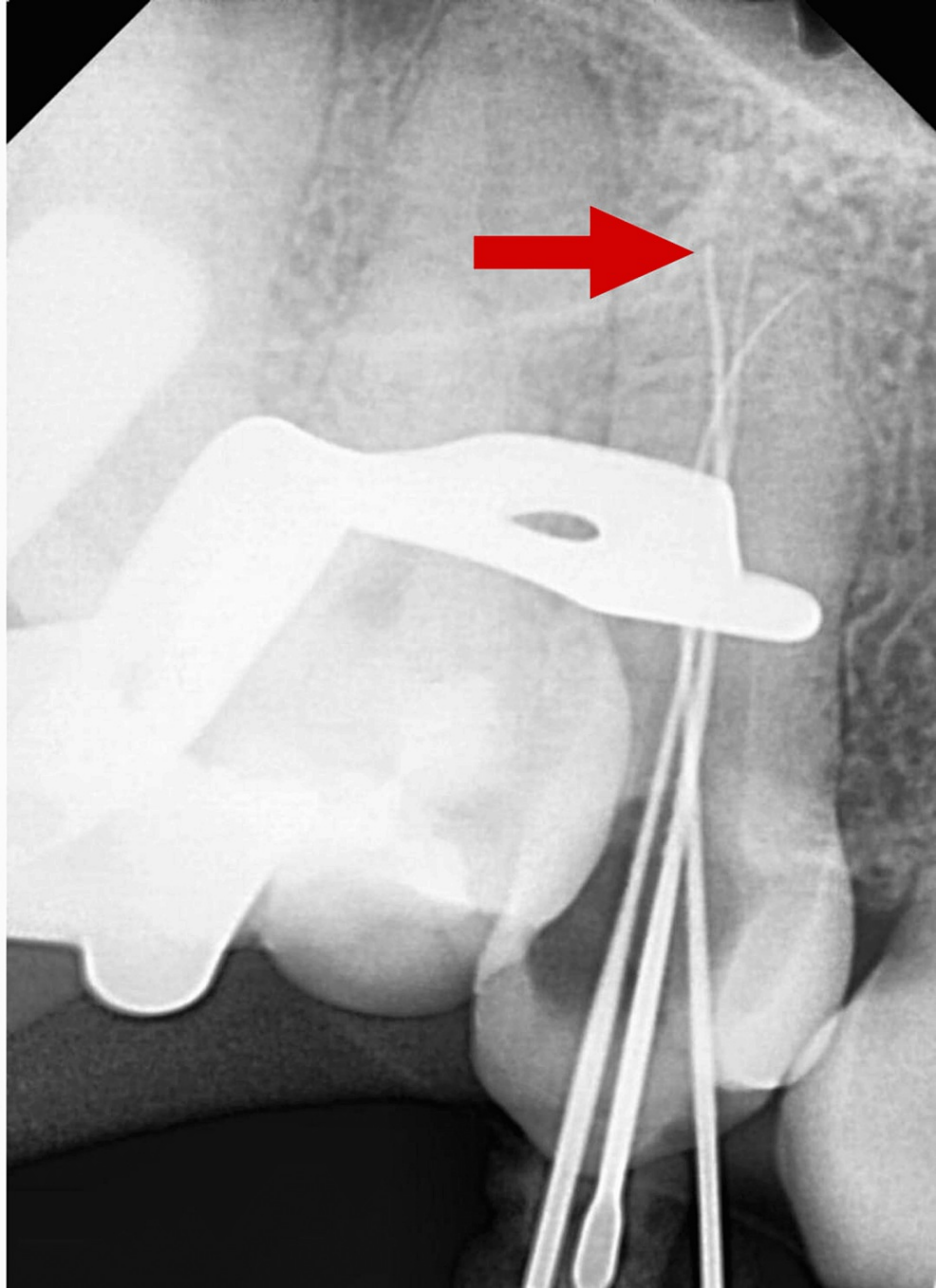
Working length radiograph Arrow depicting distobuccal canal

A No. 10 K-file (Dentsply Maillefer, Ballaigues, Switzerland) was used to locate all three root canal openings and measure the working length of each canal using an electronic apex locator (Root ZX; J. Morita Inc., Osaka, Japan) (Figure 3).

**Figure 4:**
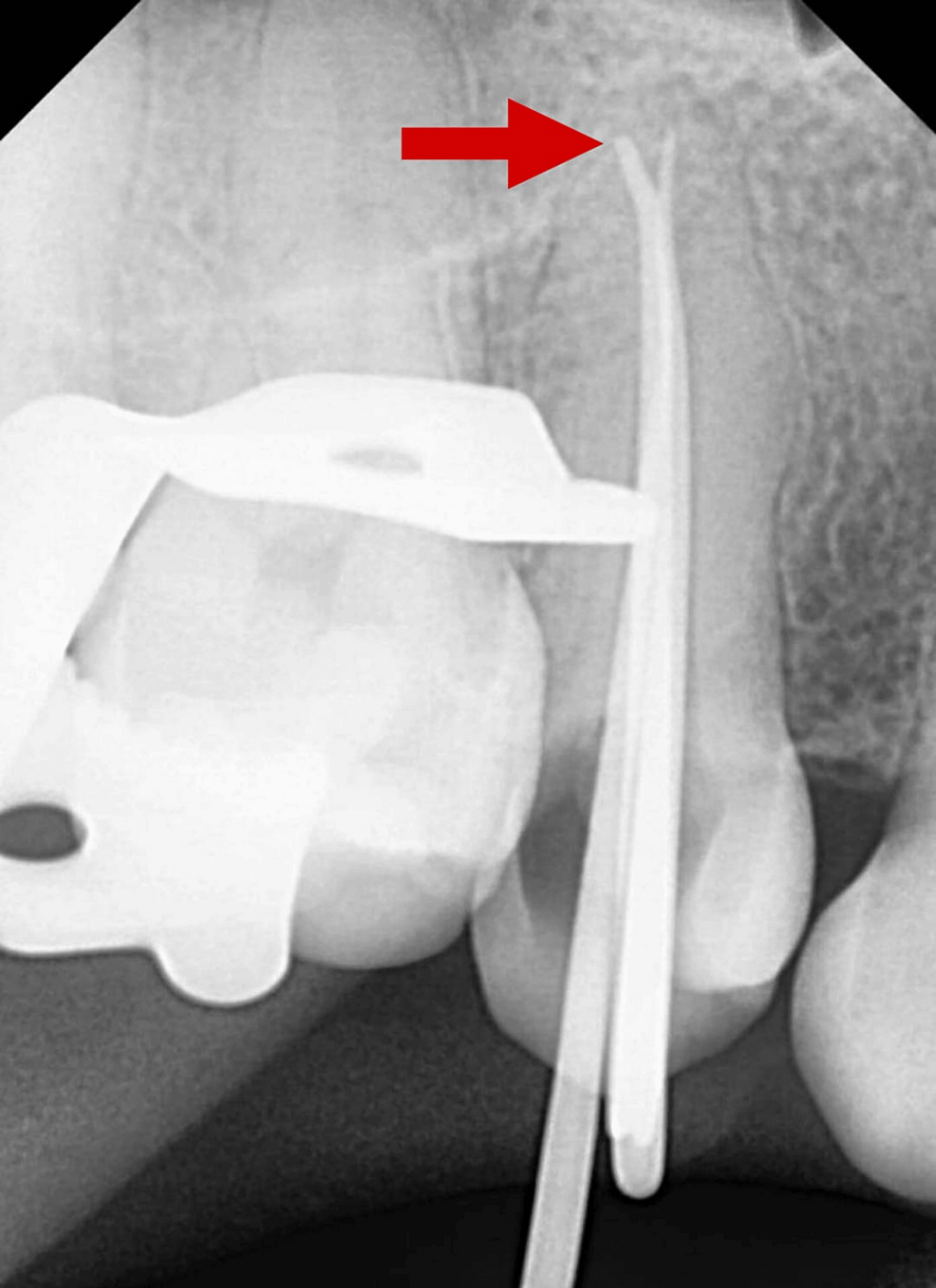
Master cone fit check Arrow depicting distobuccal canal

The biomechanical preparation of the root canals was performed using rotary Ni-Ti files (Woodpecker Ltd., Guilin, China) up to size 25 with a 6% taper in the mesiobuccal, distobuccal, and palatal canals. The canal was irrigated using 3% NaOCl (Parcan N; Septodont, Saint-Maur-des-Fossés, France) and 0.9% saline (Nivy Remedies Private Limited, Hatbaria, India) alternatively. The temporary restorative material (Neotemp and Neoendo; Orikam Healthcare Private Limited, Gurgaon, India) was given and the patient was further recalled after four days. On the second appointment, the temporary restorative material was removed and all the canals were sonically activated with 17% neo ethylenediaminetetraacetic (EDTA) gel (Neoendo) using a red medium-sized tip (25/04) of sonic endo activator (Dentsply Sirona, Charlotte, USA). All the canals were then irrigated using 3% NaOCl and 0.9% saline alternatively. Following irrigation, 0.06 #25 gutta-percha master cones were placed in the root canals (Figure 4).

**Figure 5:**
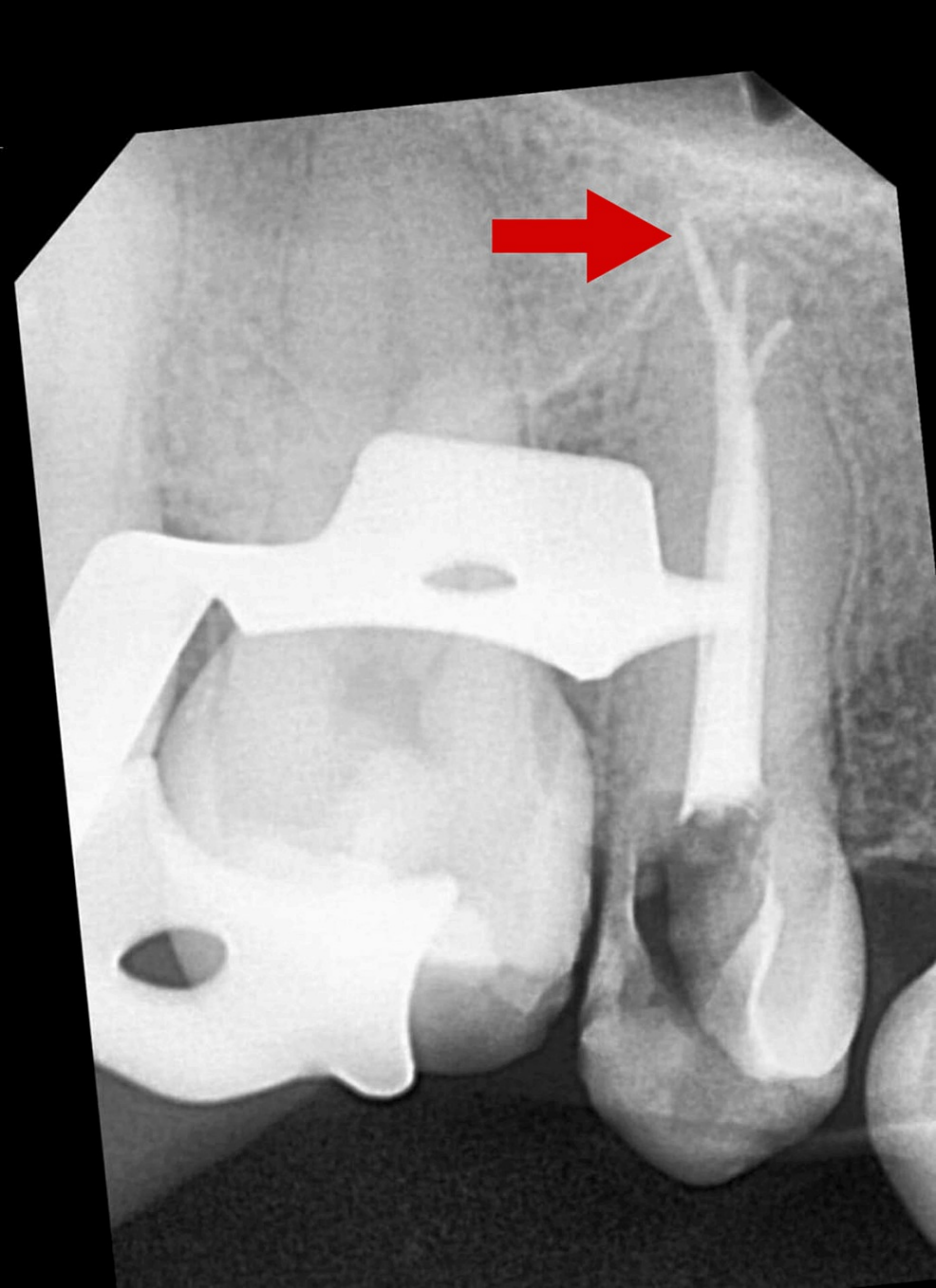
Obturation radiograph Arrow depicting distobuccal canal

Obturation was carried out with master cones and epoxy resin-based sealer (Diaproseal; DiaDent Group International, Burnaby, Canada) using the cold lateral condensation technique (Figure 5).

**Figure 6:**
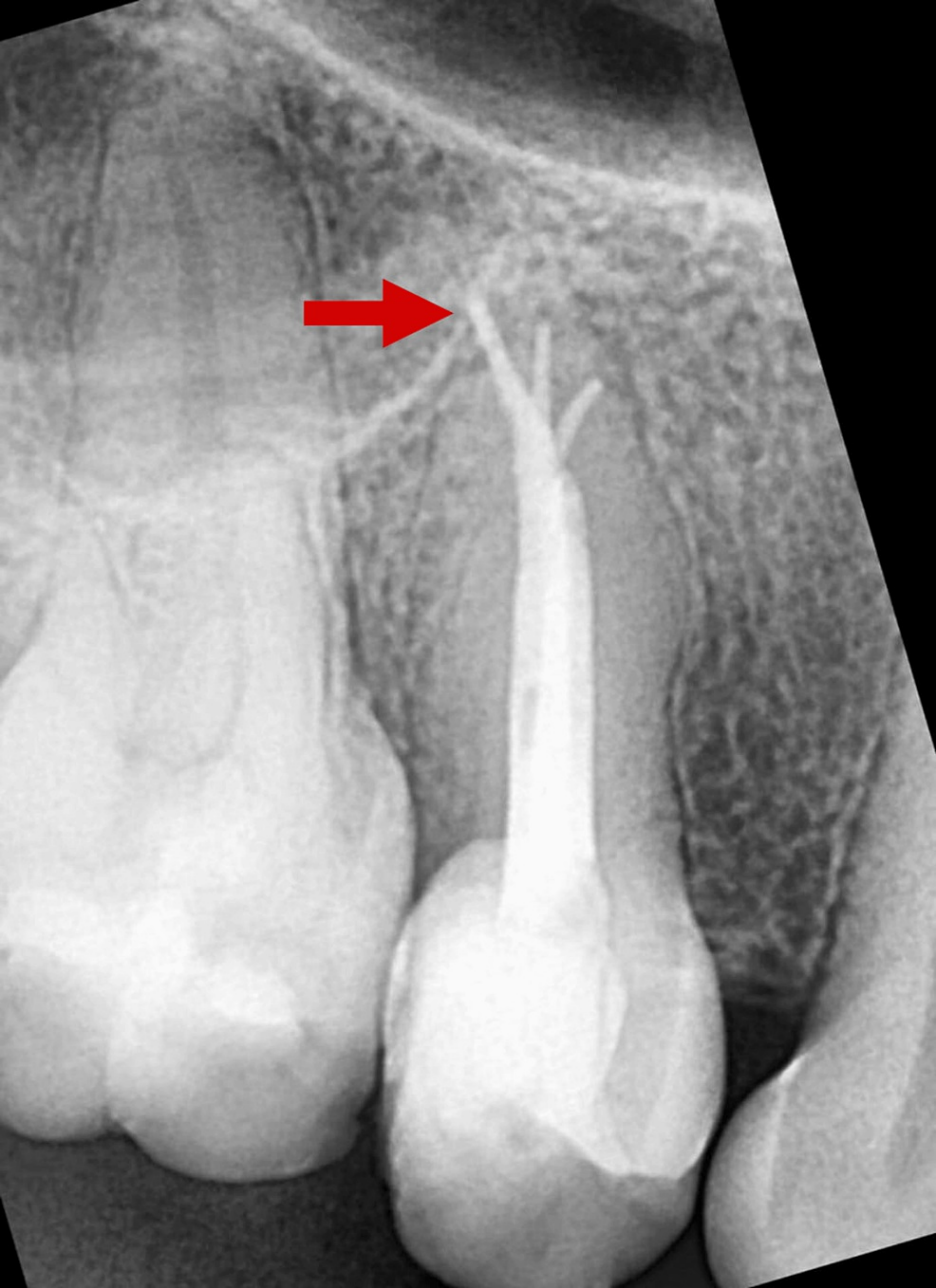
Post-endodontic restoration radiograph Arrow depicting distobuccal canal

Post-endodontic composite restoration (Spectrum; Dentsply Sirona, Charlotte, USA) was done (Figure 6).

## Discussion

Even highly skilled endodontists may find root canal treatment of maxillary first premolars challenging due to the anatomical variations they exhibit. These variations include the presence of multiple roots, variable numbers of canals, diverse configurations of canals and pulp chambers, as well as longitudinal and directional depressions on the root surfaces. Additionally, visualizing the apical anatomy on radiographs can be difficult, adding to the complexity of treatment planning and execution [14].

According to Krasner and Rankow [15], laws of the access opening or nine guidelines are followed by more than 95% of clinicians, whereas various morphological variations such as the tubercle on the occlusal surface of premolars or molars, which may contain pulp tissue, increase the risk of pulp exposure. The upper second premolar typically features one or two canals. If a third canal is present, as is the case with the upper second molar, it is considered a variation [16]. Magnification tools, such as dental operating microscopes or loupes, enhance the clinician's ability to visualize the intricate details of the tooth structure, including the pulp chamber and root canal system. This improved visualization is crucial for detecting small orifices and subtle anatomical variations that indicate the presence of an extra canal. In the present case, 3.5x dental loupes (Zumax Medical Co., Ltd., Suzhou, China) are used for magnification and illumination purposes. For detection of the additional root and its canals, correct evaluation of the X-rays that are taken preoperatively should be done [17]. It is usually advised to take more radiographs from various angulations like mesial and distal to get extra knowledge if some morphological aberrations are assumed while taking X-rays. The significance of the SLOB rule (Same Lingual, Opposite Buccal) in radiography for detecting extra canals in premolars is paramount for accurate endodontic diagnosis and treatment. By applying the SLOB rule, clinicians can take two radiographs at different angulations to ascertain the exact position of suspected additional canals. When the X-ray tube is shifted, observing the movement of the canal relative to the shift allows the dentist to determine whether the canal is located on the lingual or buccal side. The term ‘small molars’ or ‘radiculous’ is given to the upper second premolar if it has three roots and three canals (mesiobuccal, distobuccal, and palatal) same as the upper molar [18]. A basic recommendation for identifying a three-root upper premolar direct way on a preoperative radiograph is that the mesial-distal dimension of the image of the mid portion of the root is the same or more than the mesio-distal dimension of the image of the crown, the probability of having three canals in the upper second premolar increases. This recommendation is a commendable visual marker but not conclusive. For the proper location of the openings of the root canals having the most intricate morphology, minor changes in the preparation of the access cavity are needed [19]. In the case of upper premolars having three roots, the outline form of the access cavity should be T-shaped for locating the openings of the root canal appropriately [20].

The obturation of a maxillary second molar with three canals presents significant challenges due to the complex anatomy and technical demands involved. The canals may be narrow, curved, or branch at unusual angles, making it difficult to negotiate and prepare them adequately. Additionally, managing the obturation materials in three separate canals simultaneously can be demanding, with risks of overfilling or underfilling, which can compromise the seal. The success rate of the warm vertical compaction and cold lateral compaction obturating techniques has no difference [21]. The most frequently used obturating technique is the cold lateral compaction of gutta-percha. Schilder et al. endorsed the warm vertical condensation obturating technique [22]. All portals of the root canal system, including lateral canals and apical branches, are closed with filling material through three-dimensional obturation. However, warm vertical compaction is technique-sensitive and requires specialized equipment and significant skill, whereas the cold lateral compaction method is more straightforward, requiring less specialized equipment and posing a minimal risk of thermal damage to surrounding tissues [23]. Accurate diagnosis and thorough clinical and X-ray examination are the key factors in successful endodontic treatment. Cone beam computed tomography (CBCT) allows for precise visualization of the tooth's internal structure, identifying the exact location, curvature, and configuration of all canals, including the often-missed third canal. This advanced imaging technology aids in better access cavity preparation, thorough cleaning, shaping, and effective obturation of all canals, thereby improving the overall success rate of endodontic treatments [24]. Besides having an in-depth understanding of the typical structure of the canals of the root, the dentist must also keep in mind that there may be dissimilarities in the anatomy of every tooth. So, extra caution should be taken. The doctor must be mindful of the potential differences between teeth and exercise caution when performing a root canal treatment, in addition to understanding the normal anatomy.

## Conclusions

The presence of three canals in the upper second premolar underscores the complexity of its root canal anatomy. Clinicians must stay vigilant, embrace this variability, and utilize advanced diagnostic and treatment techniques to manage these anatomical variations and provide optimal patient care. Access cavity preparation should be performed with precision to locate all canal orifices, and thorough cleaning and shaping of all canals are essential to eliminate bacterial pathogens and prevent reinfection. Together, magnification and CBCT form a powerful combination in endodontics, especially for challenging cases like a maxillary second premolar with three canals. Magnification ensures meticulous procedural execution, while CBCT provides a thorough diagnostic foundation, ultimately leading to higher success rates and better patient outcomes. Continued research and clinical experience are essential for further advancing our understanding of this intriguing phenomenon and refining treatment protocols to achieve consistently successful outcomes.
